# Husserl, the mathematization of nature, and the informational reconstruction of quantum theory

**DOI:** 10.1007/s11007-020-09523-8

**Published:** 2020-12-03

**Authors:** Philipp Berghofer, Philip Goyal, Harald A. Wiltsche

**Affiliations:** 1 Department of Philosophy, University of Graz, Graz, Austria; 2 Department of Physics, University at Albany (SUNY), Albany, NY USA; 3 Department of Culture and Society (IKOS), Unit for Philosophy and Applied Ethics, Linköping University, Linköping, Sweden

**Keywords:** Phenomenology, Philosophy of physics, Quantum theory, Informational reconstruction of quantum theory, Mathematization, Edmund Husserl, The Crisis of European sciences

## Abstract

As is well known, the late Husserl warned against the dangers of reifying and objectifying the mathematical models that operate at the heart of our physical theories. Although Husserl’s worries were mainly directed at Galilean physics, the first aim of our paper is to show that many of his critical arguments are no less relevant today. By addressing the formalism and current interpretations of quantum theory, we illustrate how topics surrounding the mathematization of nature come to the fore naturally. Our second aim is to consider the program of *reconstructing* quantum theory, a program that currently enjoys popularity in the field of quantum foundations. We will conclude by arguing that, seen from this vantage point, certain insights delivered by phenomenology and quantum theory regarding perspectivity are remarkably concordant. Our overall hope with this paper is to show that there is much room for mutual learning between phenomenology and modern physics.

## Introduction

It is no overstatement to say that Husserl’s last major publication *The Crisis of European Sciences and Transcendental Phenomenology* is a key text in twentieth century philosophy of science. In it, Husserl offers a thorough analysis of what he considered to be a deeply-rooted “crisis of our culture and the role here ascribed to the sciences.”^1^ Part of this crisis is that science has lost touch with the realities of the proverbial “man on the street” and thus fails to answer the most pressing questions, “questions of the meaning and meaninglessness of the whole of [...] human existence.”^2^ It is crucial to see, however, that Husserl’s criticism amounts to much more than a general lament about practical, cultural, political, and existential-philosophical issues surrounding modern scientific culture. On Husserl’s view, the crisis diagnosed by him is rather a direct consequence of our *theoretical* inability to come up with a single, coherent, and philosophically satisfying interpretation of the kind of scientific theorizing that followed the pioneering works of seventeenth century revolutionaries such as Galileo Galilei or Isaac Newton. Instrumental to the overall argument in the *Crisis* is what Husserl refers to as *mathematization*, i.e. the cognitive process that allows us to turn reality into a “mathematical manifold.”^3^ The aim of this paper is to argue that, although Husserl does nowhere explicitly deal with twentieth century physics, his description and critical analysis of mathematization can serve as a fruitful framework for interpretational questions about quantum physics.

The structure of our paper is as follow: In the next section we will start out with giving a somewhat more detailed presentation of Husserl’s original take on mathematization in the *Crisis*. In Sect. 3 we will take a closer look at the formalism of quantum theory. We will also discuss some conventional interpretations before introducing the program of informational reconstruction of quantum theory in Sect. 4, and describing some of the interpretative insights to which this leads. We will finally argue in Sect. 5 that reconstructing quantum mechanics on the basis of fundamental informational principles is in harmony with several key insights from Husserlian phenomenology.

## Husserl on mathematization

The term “mathematization” is coined by Husserl to denote the cognitive process through which nature is turned into a mathematical manifold. What this means, concretely, is best understood through Husserl’s interpretation of the works of the “father of modern science,” Galileo Galilei. On Husserl’s view, Galileo marks a watershed in the history of physics not primarily because of any of his individual theoretical or experimental accomplishments. What sets Galileo apart from the tradition before him is rather the larger methodological vision “that trying to deal with physical problems without geometry is attempting the impossible.”^4^ Following Husserl’s reading, this pronouncement is not just a pragmatic appeal to accept mathematics as a convenient tool of calculation or a universal medium of communication. Galileo’s amalgamation of physics and mathematics is rather the result of the underlying metaphysical premise “that *everything* which manifests itself as real [...] must have its *mathematical index,*”^5^ and must therefore be translatable into the language of geometry. By thus elevating mathematical expressibility into a criterion for what can count as objectively real, Galileo introduced an intellectual innovation that had a formative influence on the ensuing scientific worldview.

Rather than being just a convenient liaison, the intimate linkage between physics and mathematics in Galilean science is metaphysically motivated: If physics is concerned with delivering the one true and complete representation of objective reality, and if, furthermore, mathematical expressibility is the only criterion for determining what can count as objectively real, then mathematics is indeed indispensable to physics. It is crucial to note, however, that the Galilean vision resulted in several problems, the issue of “specific sensible qualities”^6^ being one of them. The problem, in a nutshell, is this: Of all the qualities through which we perceive reality, only some (namely, *primary* qualities such as shape, size, position, or number) meet the demand for mathematical expressibility quite naturally. Other qualities (namely, *secondary* qualities such as color, odor, taste, or warmth) resist any effort to be directly translated into the language of mathematics. This raises a natural question: Does the mathematical inexpressibility of secondary qualities indicate that the quest of mathematized physics to provide us with a complete representation of objective reality is unrealisable after all? Or is there a way to reconcile secondary qualities with the view that everything objectively existing lends itself to direct translatability into mathematical terms?

As is well known, Galileo opted for the second alternative and took the untranslatability of secondary qualities as a clear sign for their inexistence. On his view, “tastes, odors, colors, etc. [...] are nothing but empty names [that] inhere only in the sensible body.”^7^ Consequently, “if one takes away ears, tongues, and noses, there [...] remain the shapes, numbers, and motions, but not the odors, tastes, or sounds.”^8^ At first sight, Galileo’s proposal still seems to leave us with an incomplete picture of reality, which, after all, manifests itself through primary *and* secondary qualities. However, since its inception by Galileo, the modern scientific worldview is tied to the promise that secondary qualities can be indirectly accounted for by way of reduction to primary qualities: color can be indirectly mathematized by reducing it to wavelengths; warmth can be indirectly mathematized by reducing it to motions of electrons, atoms, and molecules. By the nineteenth century at the latest, this partial substitution of mathematical constructions for the immediately experienceable world of secondary properties had given way to a view of reality where primary qualities are no more indicative of objective existence than secondary ones.^9^ What remains is a mathematical formalism that claims to be a complete representation of objective reality, but that at the same time is entirely disconnected from the world which we directly experience.

Galileo is in the limelight of the *Crisis* because it is his distinction between primary and secondary qualities that, according to Husserl, started to drive a wedge between the life-world of pre-scientific experience and the “world of science.” What we are left with is a construal of reality in which the life-world is degraded to the status of a mere illusion, while the “real world”—the world of which science speaks through its models—is forever put beyond our experiential grasp. Yet, building on Husserl’s argument in the *Crisis*, there are two main problems with this view. First, there is a rather straightforward worry that concerns the justificatory basis of science: Although Husserl was well aware of how long-winding and intricate the relations between theory and supporting data can be, he was nevertheless very clear on the fact that “the inductive judgments [of the] exact objective sciences, by means of which we go beyond the immediately experienced to make claims about the non-experienced, are always dependent on their ultimate legitimizing basis, on the immediate data of experience.”^10^ However, if this is the case—if even the most abstract scientific theory must find its ultimate “self-evident grounding”^11^ in simple life-world experiences—, then the aforementioned construal of the relationship between life-world and science indeed borders on the self-contradictory: To claim on scientific grounds that the entire life-world is nothing but an illusion is, epistemologically speaking, to cut off the branch on which one is sitting.

The second problem concerns a fundamental naïveté which, on Husserl’s view, was already part of Galileo’s original project and later carried over into many instantiations of modern physics. The problem can be summarized as follows: Much of Galileo’s rhetoric aims at presenting his mathematical models as truthful representations of objective reality. Looking at his actual scientific practice, however, a rather different picture emerges: Considering Galileo’s theory of projectile motion, for instance, it can easily be shown that his highly idealized models represent the intended target systems neither in a purely instrumental-predictive^12^, nor in a more robust correspondence-theoretical sense.^13^ This, however, does not diminish the true value of Galileo’s accomplishment: What one learns from Galileo’s theory is not primarily how real projectiles move in Earth’s gravitational field. Following Husserl’s interpretation of Galileo, and building on the detailed accounts of historians of physics like Maurice Clavelin or Alexandre Koyré, Galileo’s true achievement rather lies in the discovery of a *new way of constituting reality*, a way that crucially depends on the ability to view nature through the lenses of mathematical models.^14^ It is this aspect which is of particular relevance from a phenomenological point of view.^15^

Since they are composed of geometrical objects such as lines, planes, or circles, Galileo’s models represent ideal limiting cases that are nowhere to be found in empirical reality. However, instead of treating these models and their components as idealities that are abstracted *from* basic life-world experiences, the very point of Galilean science is to reverse the order and let the models become prescriptive *for* how the life-world is perceived. Having mastered Galilean mechanics, then, does not primarily mean to have acquired a particular set of theories or techniques. It means, much more fundamentally, to perceive actual observable instances of flying arrows, spears, and stones *as mere approximations to the ideal case that is stipulated by the mathematical model*. Accordingly, mathematization is not merely the process of translating empirical objects, events, or processes into mathematical terms. To mathematize nature is rather to intend empirical objects, events, or processes through ideal mathematical contents and to let these idealities become the prescriptive standard for what becomes actually present to us in the realm of simple life-world experience.^16^

It is crucial to see that Husserl nowhere criticised the practice of mathematization *per se*: Husserl was clearly aware that the great success story of modern science would have been impossible without Galileo’s amalgamation of mathematics and physics. The aim of the *Crisis* is rather to turn our awareness to a host of implicit presuppositions that underlie the cognitive process of mathematization—implicit presuppositions that remained hidden from Galileo’s view and that keep shaping the mindset of modern physics ever since. One such presupposition concerns the aforementioned prescriptive role mathematical models play in the physical constitution of reality. But closely related to this are presuppositions regarding the non-perspectivity and the subject-independence of physical knowledge.^17^ Once life-world occurrences are perceived as mere approximations to the ideal limiting case that is stipulated by the mathematical model, it becomes tempting to project features of the model back onto nature itself, and thus to “take for *true being* what is actually a *method*.”^18^ Taking the apparent subject-independence and non-perspectivity of mathematical idealities as a cue, this then means to elevate these features to essential characteristics of the very concept of physical being. And once nature is constituted in this manner, further presuppositions suggest themselves rather naturally: real measurements are conceived as approximations to ideal measurements which would leave the objectively existing physical system *undisturbed*; it is assumed that ideal measurements could in principle yield *complete* information about all properties of any system; and since mathematical models can in principle encode complete information about any physical system, it is taken for granted that knowledge about all temporarily subsequent states of the system can be inferred from the initial state with *certainty*, thus supporting *determinism*. It is these three presuppositions—determinism, non-disturbance, and completeness—to which we shall return in the ensuing sections.

## Quantum theory and its challenge to Galileo’s mechanico-geometric ideal

As we have seen, one of Husserl’s principal aims in the *Crisis* is to oppose the Galilean vision on which (a) reality is exhausted by what can be mathematically captured and (b) the life-world is degraded to a mere illusion. One might wonder, however, why we should still bother with Husserl’s worries given that in the meanwhile Galilean mechanics has been superseded by quantum mechanics. Furthermore, one might get the impression that phenomenology and physics are rival projects, advocating inconsistent worldviews. In this paper, and in line of a handful of earlier works^19^, we argue for the opposite. By addressing the quantum formalism and current interpretations of quantum theory, we point out that Husserl’s worries are still relevant. By discussing the program of reconstructing quantum theory, we show that the insights delivered by phenomenology and quantum theory are surprisingly similar.

In Sect. 3.1, we introduce the quantum formalism and discuss what it means to *interpret* quantum theory. We shall see that here topics surrounding the mathematization of nature come to the fore naturally. Indeed, prominent positions reify or objectify the mathematics we use in quantum theory and explicitly argue that the three-dimensional physical space of our everyday experiences is a mere illusion. This means that Husserl’s worries, originally raised against Galileo, are still relevant today.

In Sect. 3.2, we shed light on the program of *reconstructing* quantum theory. This reconstructive program enjoys popularity in the part of the physics community working on foundations of physics. In philosophy of physics, unfortunately, the reconstructive program is largely ignored.

In Sect. 4, we interpret informational reconstructions of quantum theory and contrast the conception of *measurement* as it emerges from our reconstruction with the Galilean ideal pursued in classical mechanics. Here we see that the picture offered by the reconstructive program resonates well with phenomenological approaches to science and physics. These similarities are discussed in Sect. 5, focusing on the perspectival character of perceptual experiences and quantum measurements. Here we shall see that there are profound and systematically significant similarities between insights delivered by phenomenology and quantum theory (at least if informationally reconstructed). This means that although phenomenology and physics are usually considered entirely different projects that could not possibly benefit from each other and advocate conflicting world views, the opposite might be the case.

### Interpretation of quantum theory

The mathematical formalism of quantum theory departs in fundamental ways from the formalisms of the primary theories of classical physics (chiefly classical mechanics and electromagnetism). For example, the quantum formalism predicts that a measurement performed on a physical system will, in general, *change* the quantum state of the system. Here, “quantum state” refers to the mathematical object—a vector in a complex vector space—which, insofar as predictive use of the formalism by an experimenter is concerned, *represents* the physical state of the system.^20^ Similarly, “measurement” is a primitive term in the formalism, and appeals to the experimental physicist’s intuitive understanding of what “measurement” consists in.

Moreover, the formalism only predicts the *probability* that a specific measurement outcome will be obtained, even if the initial quantum state of the system is exactly specified.

Thus, as these two examples attest, quantum theory appears to depart from key ideals in Galileo’s conception of a mathematical theory of natural phenomena—first, that an ideal measurement allows observation without causing disturbance^21^ of the mathematical object that describes the physical state of the system; second, that an ideal theory allows prediction of *precisely* what will happen when a measurement is performed on a system given the initial conditions.

Consequently, since its creation in the 1920s, there has been an ongoing effort to understand what lessons should be drawn from the non-classical nature of the quantum formalism. That is: insofar as the theories of classical physics are an empirically-successful *formal expression* of Galileo’s vision of the possibility of a mechanico-geometrical mathematical theory of natural phenomena, what should we conclude from the fact that quantum theory—which was formulated in response to the inability to construct models based on classical physics to account for basic physical phenomena (such as the blackbody radiation curve)—does not conform with key aspects of Galileo’s vision?

The efforts that have been made to answer the above-mentioned question fall under the banner of the *interpretation of quantum theory*. Ideally, a full-blown interpretation of quantum theory would provide a coherent conceptual framework within which each non-classical aspect of the quantum formalism is rendered intelligible rather than puzzling or counter-intuitive. In doing so, such an interpretation should allow us to clearly understand how and why certain aspects of Galileo’s mechanico-geometric conception of physical reality can be retained in the face of quantum theory, while others have to be given up or modified.

Take, for example, the probabilistic nature of quantum predictions. One could interpret this fact as a consequence that quantum theory fails to take into account some pertinent, but hitherto unknown, information about the actual physical state of the system. One could then hypothesize that exact prediction *would* be possible if that additional information were available, and one might further hypothesize the nature of that additional information. Thus, one might stand behind the deterministic ideal in the face of the apparently contrary evidence supplied by quantum theory. Such an option is taken in the so-called *de Broglie–Bohm* interpretation of quantum theory, which posits that the complete description of, say, an electron requires not only a quantum state (as posited by quantum theory), but *also* the position of a localized object or “particle.” This interpretation then posits a specific equation (the so-called *guidance equation*) which governs the deterministic evolution of the particle in response to the quantum state.^22^

Alternatively, one could interpret the probabilistic nature of quantum predictions as the consequence of a *fundamental limitation* on *any* mathematical theory of nature. One could then give an account which would explain why this limitation was obscured during the heyday of classical physics owing to its limited scope, why it emerged when attention was drawn to the microscopic realm, and, most importantly, precisely what—if not missing information—is the ultimate origin of this limitation. For example, in some of his writings on the interpretation of quantum theory, Bohr argues that measurement is an inherently invasive process involving an uncontrollable change in the system under observation, and that this forces the renunciation of the ideal of non-probabilistic prediction.^23^

A major challenge faced by any putative interpretation of quantum theory is that quantum theory appears to be odds with *so many* distinct aspects of the mechanico-geometric ideal, and thus an interpretation must, ideally, simultaneously resolve many distinct points of tension. Some of these points of tension—such as the disturbance of system state by measurement, or the probabilistic nature of measurement outcomes—were evident as soon as quantum theory received its first formal expression. But others took some time to surface.

For example, in the mid-1930s, Schrödinger pointed out that, according to the quantum formalismé, the quantum state of a two-body system is, in general, *not* (as presumed in classical physics) a simple list of the quantum states of each body, but is rather a new entity in its own right—a so-called *entangled* state.^24^ Schrödinger showed that a measurement on one component of a system that had been placed in such a state would, in general, change the state of the *other* component, no matter how distant, with the nature of this change being partly determined by the specific *choice* of measurement. Some thirty years later, Bell showed that, modulo specific, non-trivial assumptions^25^ about the nature of the physical reality, these entangled states allow for quantum predictions that are *incompatible* with the classical ideal of *locality*, namely the ideal that two bodies interact with one each other via influences that propagate from one to the other at *finite* speed.^26^

The major limitation of every extant interpretation of quantum theory is that none is able to provide a coherent interpretation of *all* of the non-classical aspects of quantum theory. Indeed, most only attempt to interpret a small number of these non-classical aspects. This has given rise to a plethora of interpretations—Bohr’s interpretation, the de Broglie–Bohm interpretation, the many-worlds interpretation, to name but three—which propose radically different resolutions of the conundrum posed by quantum theory.^27^ Since interpretations are, by their very nature, one step removed from the physical theory *per se*, they are not readily open to empirical test. Hence, we are today left in the undesirable position of having many distinct interpretations on the table, each plausible to some extent yet limited in its explanatory capacity, but with no decisive way to choose between them.

A further problem that plagues specifically *realist* interpretations of the quantum state—such as the de Broglie–Bohm interpretation—is that they are in danger of implying an *implausible reification of the mathematical concepts*^28^. In quantum mechanics, the quantum state is represented by the so-called wave function. Mathematically speaking, *wave functions are vectors in a Hilbert space*. This is often expressed by saying that “[w]ave functions live in Hilbert space.”^29^ A Hilbert space is an abstract mathematical concept, namely a complete vector space on which an inner product is defined. But if the wave function is something real, does this mean that mathematical Hilbert space is physically real too?

We see now that Husserl’s worries about mathematization are still relevant and how this topic emerges in quantum mechanics. Wave functions representing quantum states live in Hilbert space. But what about tables and chairs, and you and I? What is the relationship between abstract mathematical spaces and the space we actually live in? In this context, the most straightforward reification of mathematics would occur by reifying Hilbert space. And indeed, one can find prominent voices championing Hilbert space realism.^30^ However, most consider this an implausible and unwarranted hypostatization of mathematical objects and it has been pointed out that only “[v]ery few people are willing to defend Hilbert space realism in print.”^31^

A similar but more subtle form of mathematization takes place in configuration space realism, i.e., the project of reifying the 3*N*-dimensional configuration space, *N* being the number of the particles in the universe. The main proponent of this view is David Albert, who at one point considered our impression that we live in three-dimensional space “somehow flatly illusory.”^32^ Obviously, from a Husserlian perspective, such a claim is highly suspect, to say the least. Configuration space realism, often referred to as “wave function realism,” has been quite popular and has sparked much controversy. In fact, “[t]his view of the ontology of (no hidden variable) quantum mechanics has probably been the most commonly assumed in the recent literature.”^33^ Its prevalence notwithstanding, Wallace rightly remarks that “it makes the same unmotivated conceptual move as Hilbert space realism: it reifies a mathematical space without any particular justification.”^34^

This is not the place to discuss the problems of such mathematizations in detail, but we note the Husserlian idea that no matter how abstract our scientific theories are, their justification, ultimately, lies in ordinary experiences, in what is immediately given. Accordingly, as discussed in Sect. 2, with Husserl we might warn that an interpretation of a scientific theory should not cut off the branch on which science is sitting. And indeed, this worry has been raised against Albert’s configuration space realism when it is questioned whether it can be *empirically coherent*.^35^

Concerning the formal and technical apparatus of the mathematical sciences, Husserl warned us not to be “misled into taking these formulae and their formula-meaning for the true being of nature itself.”^36^ We see how this problem also arises in quantum mechanics via wave function realism. The most common realist interpretations of quantum mechanics—the many-worlds interpretation, Bohmian mechanics, and GRW theory—are all in danger of leading to a mathematization of nature that is not based on physical principles but on mathematical formalism. Critical voices have pointed out that in these interpretations “the strategy has been to reify or objectify all the mathematical symbols of the theory and then explore whatever comes of the move.”^37^

Here we want to take a different approach. Instead of reifying mathematical constructs, the idea is to formulate physically meaningful postulates from which the quantum formalism can be derived or reconstructed. This is the program of *reconstructing* quantum theory. Proponents of this program emphasize that this basic idea of deriving the formalism from physical postulates is successfully realized in other physical theories, special relativity being the prime example:

Textbook postulates such as ‘a physical system is described by a complex Hilbert space,’ ‘pure states are described by unit vectors,’ ‘outcome probabilities are given by the Born rule,’ and ‘systems combine by the tensor product rule’ are now regarded as abstract mathematical statements in need of a more fundamental explanation. Such an explanation would be akin in spirit to Einstein’s derivation of the Lorentz transformations from the light postulate and the principle of relativity.^38^

This is a project of “taking a more physical and less mathematical approach”^39^ and attempting “to reduce the mathematical structure of quantum mechanics to some crisp physical statements.”^40^ This less mathematical but more physical approach resonates well with our Husserlian sentiments expressed above. What is more, as we shall see below, the reconstructive program leads to a picture of physics and reality that shares profound similarities with phenomenological teachings concerning the structure of experience, the epistemological significance of experiences, and the irreducibility of the subject.

### Reconstruction of quantum theory

One of the major barriers that one faces in any attempt to interpret quantum theory is that the only precise expression of quantum theory is an abstract mathematical formalism which is far removed from ordinary experience. For example, whereas classical mechanics posits that the state of a particle consists in its position and velocity, quantum mechanics describes its state as a vector in a complex-valued, infinite-dimensional vector space.

The sheer remoteness of the quantum formalism from our direct physical experience presents a formidable barrier to its conceptual assimilation. To emphasize this point: the mathematical formalism of classical mechanics was, to a considerable extent, a formal expression of *physical principles*, such as Galileo’s principle of relativity and Descartes’ principle of conservation of motion^41^. Each of these principles can be quite readily grasped, quite independently of any mathematical formulation. Moreover, each can be viewed as instances of even more basic notions—conservation as an instance of the general idea that change is underlain by changelessness; relativity as an instance of idea that, although physical observations are necessarily perspectival, there are classes of observers which are in some sense *equivalent*. In contrast, the path from ideas such as de Broglie’s wave-particle duality to Schroedinger’s equation involves many mathematical leaps (such as the introduction of complex numbers, and the transition to many-dimensional configuration space when treating a system of many particles) whose physical origin is obscure.

Removal of the above-mentioned barrier is the primary objective of the program of the *reconstruction* of quantum theory. The essential goal of the reconstruction program is to formulate *physical principles*—ideally, principles with an intuitive comprehensibility comparable to those underlying classical physics—from which the quantum formalism can demonstrably be systematically derived.

With a reconstruction of quantum theory in hand, the challenge of interpreting quantum theory shifts from the traditional interpretation of the *quantum formalism as given* to an interpretation of the *conceptual framework* (with its background assumptions) and the *principles* used in the reconstruction.

#### Informational reconstruction of quantum theory

Over the last two decades, a number of reconstructions of the core formalism of quantum theory—specifically, the Dirac–von Neumann axioms for finite-dimensional systems, and the tensor product rule for handling composite systems^42^—have been put forward.^43^

*Operational framework*

While these reconstructions differ greatly in their background assumptions and principles, most derive quantum theory within an *operational* framework. This amounts to taking as primitive the notion of *measurement*, as well the notion of an *agent* (or *experimenter*) who is capable of freely choosing which measurement to make and when (if at all) to make it. Thus, a typical reconstruction imagines that a *physical system* enters an *experiment* where it undergoes a *measurement* (which yields a particular *outcome*), is then subject to some kind of *interaction* with an apparatus before undergoing a final measurement. The italicized terms—“experiment,” “physical system,” “measurement,” “outcome,” and “interaction” are all technical terms that are taken as primitive. The choice of which measurements are performed, and which interaction is enacted, is left to the agent.

This operational framework is not devoid of implicit assumptions, which could legitimately be questioned. For example, the notion of a “physical system” upon which an experiment is performed presumes that there is *something* which persists (retains its identity) during the course of the experiment, a nontrivial assumption given the measurements and interactions that are taking place. Similarly, it is implicitly assumed that the agent’s choice is independent of the physical system itself. Nevertheless, such notions are taken as primitive in the *practice* of physics, whatever be the theory under consideration—every physical theory is, in practice, ultimately developed and tested on a laboratory workbench. Hence, to question the assumptions underlying the operational framework would be tantamount to questioning our basic conceptualization of what happens on the laboratory workbench—which, after all, is the closest we come to the life-world in the context of quantum theory.^44^

*Informational view of physical theory*

Most of the recent reconstructions of quantum theory that utilize an operational framework *also* adopt an *informational view* concerning physical theory.^45^ According to this view, measurement is a means to gather data about the “physical world,” and thus provides information *about* the physical world. A physical theory is accordingly, in essence, a compact codification of the regularities that we discover in that information. This view resembles Mach’s view that a physical theory is, above all, an economical codification of the regularities extracted from sense data. But the informational view typically stops short of incorporating the extreme instrumentalism that is often associated with Mach’s thinking.

The informational view accordingly regards the conceptual framework and specific assumptions of a physical theory as—paraphrasing the words of Wheeler^46^—a kind of papier-mâché that we fill in between the “iron posts” of measurement outcomes.

Accordingly, an informational reconstruction typically eschews manifestly mechanical or geometric models of physical systems. Instead, its assumptions are *informational* in nature. For example, it is commonly posited that the state of a physical system is (insofar as the predictive use of the theory is concerned) described by a mathematical object—the mathematical state—that enables prediction of the outcome probabilities of any measurement performed upon it; and that the mathematical state contains more degrees of freedom than those that are needed to predict the outcome probabilities of any given measurement.

## Interpreting informational reconstructions of quantum theory

As stated above, one of the primary motivations of the reconstructive program is to shift the paradigm of interpretation: rather than interpreting the *quantum formalism* as given (as has been the case historically), we instead interpret the *conceptual framework and principles* employed in a given reconstruction. Whether or not such an interpretative project is fruitful for any given reconstruction depends on the perspicuity of the conceptual framework and principles.

In this connection, we recall the above-mentioned historical analogy with Einstein’s derivation of the Lorentz transformations. Prior to Einstein’s derivation, numerous attempts had been made to interpret these transformations against the background assumption that there exists a privileged reference (ether) frame by positing new hypotheses (such as Fitzgerald’s hypothesis that a body in motion through the ether is contracted in its direction of motion). However, Einstein interpreted these same transformations on the background assumption that all inertial frames are equivalent (Galileo’s principle of relativity). Although this interpretation was directly opposed to the competing one, it rapidly gained widespread assent due to the perspicuity of the conceptual framework and physical principles that formed the basis of his derivation.

The full interpretation of an informational reconstruction of quantum theory is beyond the scope of this paper. Instead, we focus on a specific interpretative issue, namely that concerned with the *perspectivity* of the quantum measurement process.

### Perspectivity of quantum measurements

The Galilean ideal asserts that it is possible to construct a mathematical theory of nature satisfying the following fundamental criteria:

1. *Determinism* It is possible, in principle, to describe a physical system with sufficient precision that it is possible to predict the outcome of a measurement performed on that system with certainty. Accordingly, in theoretical terms, given the initial (mathematical) state of the system (the "initial conditions"), the state of the system at any later time is *determined by* (i.e. is a *mathematical function of*) the initial state.
2. *Non-disturbance* Although practical measurements involve some degree of interaction with a physical object of interest, and thus unavoidably disturb the behaviour of the measured object, these practical measurements are, in fact, an approximation to *ideal* measurements that involve no interaction whatsoever, and thus do not disturb the measured object. In theoretical terms, the mathematical state is unaffected when an ideal measurement is performed on the system.
3. *Completeness* It is possible to simultaneously measure *all* of the properties of any physical system. In practice, such a measurement can be implemented by performing, in rapid succession, perspective-limited measurements (each capable of measuring only a limited number of properties), this being possible due to the non-disturbing property of measurements. In theoretical terms, there exists a single ideal measurement such that the (mathematical) state of the system is determined by the outcome of such a measurement.

In the simplest case of a classical particle moving through space, the mathematical state of the particle is given by its position and velocity, and a single idealized measurement can be performed which yields the values of these properties without changing their values.

In contrast, informational reconstructions of quantum theory^47^—imply the following:

1. *Indeterminism* Measurement outcomes are *not* determined by the quantum (mathematical) state of the system. Instead, the quantum state determines only the *probabilities* of these outcomes.^48^
  For example, given a so-called qubit (an elementary quantum system), the *state* of the system can be represented by a point, $P$, on a unit sphere, which we can represent by a unit vector, $\mathbf {r}$, from the sphere’s origin to $P$. Similarly, each possible measurement corresponds to a point on this sphere, described by unit vector, $\mathbf {n}$.
    A measurement $\mathbf {n}$ on a qubit with state $\mathbf {r}$ does not tell us what $\mathbf {r}$ is. Rather, the measurement yields one of two possible outcomes—labelled “+” and “−”—and the state determines the probabilities of these outcomes. In particular, $p_+ = (1 + \mathbf {r}\cdot \mathbf {n})/2$ is the probability of outcome “+.”
2. *Disturbance* Once any given (projective) measurement is performed on a system, its quantum state is necessarily *changed* to reflect the measurement outcome.
  If a measurement $\mathbf {n}$ is performed on a qubit that is initially in state $\mathbf {r}$, and yields outcome “+,” the state of the qubit *after* the measurement is simply $\mathbf {n}$. Thus, the post-measurement state is *determined* by the measurement vector and its outcome, and no further information about the qubit’s *initial* state, $\mathbf {r}$, can be gained by performing additional measurements on the same qubit.
3. *Non-completeness* Each (projective) measurement only provides information about one half of the predictively-relevant degrees of freedom of the quantum state. Due to the disturbance property above, the non-completeness of a measurement cannot be overcome by simply performing *another* measurement on the same system afterwards.
  For example, in the above-mentioned qubit example, a measurement, $\mathbf {n}$, made on a qubit prepared in state $\mathbf {r}$ only yields information about the *projection* of $\mathbf {r}$ on $\mathbf {n}$, namely about $\mathbf {r}\cdot \mathbf {n}$. In terms of the spherical polar angles $\theta , \phi$, of $\mathbf {r}$ relative to $\mathbf {n}$, the measurement provides information about $\theta$, but not about $\phi$.

In most informational reconstructions, indeterminism is allowed for as part of the operational framework itself, with disturbance then following as a consequence of the requirement—shared by classical theories—that measurements are *repeatable* (or *reproducible*), namely that, after a measurement has been performed on a system, its immediate repetition yields the same outcome with certainty. Non-completeness is introduced either directly as a postulate^49^ as a formalization of Bohr’s notion of *complementarity*), or indirectly via other postulates.^50^

Collectively, these three properties can be summarized as saying that quantum measurements are *perspectival*. That is, given a physical system (as described by quantum theory), one must first *choose* from a set of possible different measurements. The chosen measurement provides a distinct perspective on the system in two distinct senses. First, it only provides information about *certain* degrees of freedom of the state of the system. Second, one only receives limited information about *these* degrees of freedom. Having received this information, one cannot repeat the same measurement on the system in the hope of receiving more information about its original state, because its state is irrevocably changed by the initial measurement. Similarly, one cannot subsequently perform a *different* measurement in the hope of obtaining information about *other* degrees of freedom, because—again—the system is no longer in its original state. Thus, the agent’s choice of initial measurement is directly consequential, both in terms of what the agent does and does not learn about the system, and in how the state of the system is changed following her intervention.

## Phenomenological reflections on informational reconstructions of quantum theory

### Points of contact between phenomenology and the reconstructive program

Several features of the reconstructive program resonate well with phenomenological approaches to science and physics.

First, instead of taking the mathematical formalism of quantum theory for granted and interpreting it as given, the reconstructive program begins with inquiring what motivates the formalism in the first place. Where does the quantum formalism come from? The reconstructive program seeks to identify the basic physical principles from which the formalism can be derived or reconstructed. What is interpreted, then, is not the formalism as such but the formalism in light of the conceptual framework and underlying physical principles employed in a given reconstruction.

This claim that we must not take the mathematical formalism of a physical theory for granted but inquire as to its origin and motivation can also be found in Husserlian phenomenology. In Section 9h of the *Crisis*, entitled “The life-world as the forgotten meaning-fundament of natural science,” Husserl argues that “Galileo was himself an heir in respect to pure geometry.”^51^ This is because Galileo’s geometry was “preceded by the practical art of surveying” and this “pregeometrical achievement was a meaning-fundament for geometry, a fundament for the great invention of idealization.”^52^ For Husserl, “it was a fateful omission that Galileo did not inquire back into the original meaning-giving achievement” of what is an “idealization practiced on the world of our everyday experiences.”^53^ This omission is why it seemed obvious to Galileo that geometry could be applied to nature but “this obviousness was an illusion.”^54^ The basic idea here is that inquiring into the origin of geometry reveals that applying geometry to capture nature involves a *process of idealization*. According to Husserl, Galileo confused what is a *method to represent reality* with reality itself and this confusion was a consequence of the fact that Galileo took the mathematical-geometrical formalism that worked so well for granted and interpreted it as given.

Similarly, in the reconstructive program, we find the (implicit) criticism that many researchers working on interpretations of quantum mechanics only look at the quantum formalism as it is, try to make sense of it (which may involve more or less minor modifications of the formalism), but are not concerned with its sense-giving foundation. Of course, the difference is that Husserl considered the *life-world* the sense-giving foundation of the sciences and is interested in how the respective formalism emerges from this foundation. The reconstructive program, on the other hand, is interested in how the formalism emerges from underlying physical principles. However, the basic ideas of both approaches fit well and we believe that they may complement each other.

Second, in the *operational* framework of the reconstructive program, the *agent* and her *experiences* take center stage. The operational framework implies that, essentially, the experiences of the agent are taken as the basic raw materials out of which any worldview is build up. This is in perfect agreement with phenomenology. It is *the* core commitment of a phenomenological epistemology that knowledge is always knowledge of a *subject* and that every piece of knowledge can be traced back to *epistemically foundational experiences*.^55^ For Husserl, such justification-conferring experiences include perceptual experiences, introspective experiences, a priori intuitions, and evaluative experiences. Importantly, an experience, like any intentional act, is an “intentional relation of consciousness to object”^56^ where we have “the ego as one pole of the relation in question, while the other pole is the object”^57^ such that “[j]ust like any object-pole, the Ego-pole is a pole of identity.”^58^ This means that in phenomenology the subject is *irreducible*. Similarly, in the reconstructive program, the agent is a *primitive notion* and the *choices* of the agent are regarded to be *independent of the physical system itself*. What is more, since in the operational framework the worldview originates from the agent’s experiences, reconstructive approaches are at least skeptical towards projects of mathematizing nature. Above we have seen that, in the context of realist interpretations of the wave function, Hilbert space realism^59^ and configuration space realism^60^ are in danger of implying an empirically incoherent mathematization of nature. Similar to Galileo, they objectify or reify the mathematics used in their successful physical theories.

Third, another important aspect of the *operational* framework is that this approach is close to the *practice* of the physicist and unbiased with respect to *quantum phenomena*. We do not consider the properties of classical mechanics properties that must be preserved in quantum theory. One might argue that this is a different attitude than the one prevailing, for instance, in the de Broglie-Bohm interpretation, in which particle ontology and determinism are preserved (with some costs) by slightly modifying the quantum formalism. This is not to say that the reconstructive program is incompatible with Bohmian mechanics. However, phenomena such as complementarity, entanglement, nonlocality, and the apparently probabilistic nature of measurement outcomes tend, in the reconstructive approach, to be taken seriously without the ambition to explain them away if possible. Alluding to Husserl’s famous quip in *Ideas 1*,^61^ proponents of the reconstructive program may proclaim to be the genuine positivists because they respect the (quantum) phenomena. Furthermore, the operational framework wants to be close to the *practice* of the physicist. No rules are imposed “from above” on what measurement is or how a physicist has to perform a measurement. Instead, notions such as “measurement” or “experiment” are considered primitive notions. This is also in line with Husserl. As Mirja Hartimo puts it, Husserl “does not make a priori, metaphysical claims about the sciences. Nor is he aiming at a philosophical view of what the sciences *should* be or become like. Instead he is describing the scientific practices and their normative goals as he finds them at each point of time.”^62^

Fourth, informational reconstructions of quantum theory imply (1) the indeterminism of measurement outcomes, (2) the disturbing character of projective measurements that necessarily change the observed quantum state, (3) and the incompleteness of the information provided by measurements. This means that in quantum mechanics the physicist or agent is not and cannot be an innocent bystander that gains a complete and completely objective picture of nature. This is in agreement with the phenomenological picture according to which the subject is an embodied subject that cannot be separated from the world it acts upon. What is more, it is a core conviction of phenomenology that a purely objective view from nowhere is impossible. “There is no pure third-person perspective, just as there is no view from nowhere.”^63^ Instead, “[a]ny understanding of reality is by definition perspectival. Effacing our perspective does not bring us any closer to the world. It merely prevents us from understanding anything about the world at all.”^64^

One of Husserl’s main contributions to a proper phenomenological analysis of perceptual experience is the disclosure of what he calls the *horizontal structure of experience*. In this context, Husserl shows that perceptual experiences are genuinely *perspectival*. In the following section, we address Husserl’s conception of horizontal intentionality and shed light on similarities between the perspectival character of perceptual experiences and the perspectival character of quantum measurements.

### The perspectival character of perceptual experiences and quantum measurements

An important achievement of Husserl’s mature phenomenology is the discovery of the *horizontal structure* of intentionality. To make a long story^65^ short: As phenomenological descriptions reveal, perceptual experiences always and necessarily go beyond what is directly given. What this means can be illustrated by means of an example: Assume that you are undergoing a perceptual experience of a laptop. At first glance, what presents itself to you in experience is a three-dimensional object in space. But a more accurate description reveals that what is really sensuously given to you is not simply a laptop, but only *one single profile of the laptop*, its current frontside. To be sure, you could alter your position and make the current backside the new frontside, and vice versa. But this doesn’t change the fact that the laptop is always given *in perspectives* and that, more generally, physical things always and necessarily have more parts, functions, and properties than can be actualized in one single intentional act. The laptop-as it is intended-is *transcendent*, not only in the sense that it can be seen from indefinitely more perspectives than you can take up at a given point in time. The laptop is also transcendent in the sense that it has, for instance, a momentarily hidden internal structure, a history, certain practical functions, or many properties that aren’t in the center of attention right now.

So, a closer look at how physical things appear to us reveals that our intentions towards these things always “transcend” or “go beyond” the actual experiences that give rise to them. As the example of the laptop shows, there is a describable difference between what is meant through a particular perceptual act (the laptop in front of you) and what is sensuously given (the laptop’s facing side with its momentarily visible features). Phenomenologically construed, this discrepancy does not represent a problem that must be somehow remedied, e.g. by proposing a theory that explains how a number of seemingly disconnected profiles add up to a homogeneous thing to which we then attribute these profiles. The fact that our perceptual intentions always transcend the sphere of direct givenness is rather to be treated as a phenomenologically discoverable feature of experience itself: Intending is, as Husserl puts it, always and necessarily an “*intending-beyond-itself*.”^66^

Husserl characterizes the *perspectival* character of perception as follows:

Of necessity a physical thing can be given only ‘one-sidedly’; [...] A physical thing is necessarily given in mere ‘modes of appearance’ in which necessarily a *core of* ‘*what is actually presented*’ is apprehended as being surrounded by a horizon of ‘*co-givenness*,’ which is not givenness proper.^67^

When Husserl illuminates the perspectival character of perception, he not only stresses that perception is incomplete but also that physical objects in perception always appear from a certain point of view.

All orientation is thereby related to a null-point of orientation, or a null-thing, a function which my own body has, the body of the perceiver. And again, the perspectival mode of givenness of every perceptual thing and of each of its perceptual determinations—on the other hand, also of the entire unitary field of perception, that of the total spatial perception—is something new. The differences of perspective clearly are inseparably connected with the subjective differences of orientation and of the modes of givenness in sides.^68^

A further aspect of perception is that previous experiences shape the way we perceive. Perception is not a faculty that allows us to see the world as it is objectively, independent from our history, background beliefs, etc. To put it differently, “experience is not an opening through which a world, existing prior to all experience, shines into a room of consciousness; it is not a mere taking of something alien to consciousness into consciousness.”^69^ This aspect of perception is closely related to discussions about the theory-ladenness of perception.

Although Husserl regards experiences as a source of immediate justification, he is well aware that experiences are not windows to the world through which we see how the world is in itself thoroughly objectively. Instead, experiences present their objects in a certain way that at least partly depends on subjective factors such as previous experiences, background beliefs, etc. To put it differently, the objects we experience and think about do not have an objective sense that is for us to be discovered. Instead, we ourselves constitute the sense of the objects we engage with.

For Husserl, sense is not simply something outside us that we apprehend, it is something that is ‘constituted’ or put together by us due to our particular attitudes, presuppositions, background beliefs, values, historical horizons and so on. In short, phenomenology is a reflection on the manner in which things come to gain the kind of *sense* they have for us.^70^

Accordingly, we cannot achieve an objective view on the world, our experiences are necessarily incomplete and perspectival, by engaging with the world we constitute and thereby change the sense of the objects we encounter, and we only have limited knowledge of the present and the future. As we have seen in Sect. 5.1, all this is in contrast to the Galilean ideal pursued in classical mechanics. Of course, our everyday experiences are a different encounter with the world than scientific investigations. The fact that there is a profound discrepancy between the Husserlian picture and the Galilean ideal does not imply that one of them is mistaken. For Husserl, the discrepancy is explained by the fact that Galilean mechanics (unbeknownst to Galileo) is directed at an *idealization* of reality, not at reality itself.

In short, there are unexpected but profound and systemically significant similarities between insights delivered by phenomenology and quantum mechanics. Nevertheless, as must be expected when comparing the richness of our lived experience as understood via phenomenology to the austere and abstract formalism of a physical theory concerned with a sharply-delineated realm of experience, these similarities conceal many subtle points of tension. For example, the openness in our experience has several facets—we are rarely able to delineate the set of possible outcomes of a given action, let alone compute the probabilities of these outcomes given our prior knowledge. In contrast, the abstractions embodied in the quantum formalism apply to measurements with a definite, pre-defined number of possible outcomes. Points of dissimilarity such as this require careful attention, and may indeed suggest ways in which the formalism could be generalized.

The development of a sophisticated and nuanced account of the similarities as well as the points of tension between phenomenology and quantum theory—when viewed through the lens of the informational reconstruction program—is a challenging project that will likely require cross-disciplinary collaboration. However, broadly speaking, very little research has been conducted at the intersection of phenomenology and quantum mechanics. There are several reasons for this unfortunate lack of engagement. One reason is that in current debates one finds a plethora of rival interpretations of quantum mechanics. Accordingly, there is no consensus on what quantum mechanics tells us. We believe that the project of reconstructing quantum theory helps us to get a clearer picture. We hope that the present work shows that phenomenology in particular but also philosophy of physics in general could benefit from engaging with the reconstructive program.

## Footnotes

1 Husser (1970, p. 5).

2 Husser (1970, p. 6).

3 Husser (1970, p. 23).

4 Galilei (1967, p. 203).

5 Husser (1970, p. 37).

6 Husser (1970, §9c).

7 Galilei (2008, p. 185).

8 Galilei (2008, p. 187).

9 Weyl (1949, pp. 110–13).

10 Husserl (1973, p. 121); our translation.

11 Husser (1970, p. 123).

12 Segre (1991, pp. 94–7).

13 Wiltsche (2017, pp. 160–65).

14 According to Clavelin, “Galilean science was first of all a transition from one conceptual framework to another, [...] an unprecedented fusion of reason and reality” (Clavelin 1974, xi). Koyré sees Galileo’s main accomplishment in the introduction of a new “mental attitude, [an attitude] that is not [purely physical or] purely mathematical [but] *physico-mathematical*” (Koyré 1978, 108).

15 Cf. Islami and Wiltsche (2020).

16 Although much more could be said on this issue, even these brief remarks should suffice to show that there are interesting relations between Husserl’s late philosophy of science and the extensive literature on models and modeling in contemporary “mainstream” philosophy of science (cf., for an introduction to the latter, e.g. Morgan and Morrison 1999; Bailer-Jones 2009; Gelfert 2016; Frigg and Hartmann 2020). In our view, the most distinct feature of a genuinely phenomenological account on scientific modeling is the claim that models should not primarily be seen as representational vehicles but rather as cognitive filters that normatively guide the way in which reality is constituted. For more detailed discussions of this, cf., e.g., Wiltsche (2019), Islami and Wiltsche (2020) and Wiltsche (forthcoming).

17 As we will see below, certain developments in modern physics, particularly in quantum mechanics, cast doubt on non-perspectivity and subject-independence. We will show that the formalism of quantum theory, particularly when informationally reconstructed, exhibits a number of features and implications that are surprisingly consistent with phenomenological teachings. However, we do not want to suggest that Husserlian phenomenology is the only philosophical framework that has this virtue. In particular scientific perspectivism, an approach recently developed by Giere (2006) and Massimi (2012) and Massimi (2018), seems very promising in this respect. In fact, we believe that there are substantial similarities between our phenomenological approach and perspectivism. For a detailed discussion of perspectivist elements in Husserlian phenomenology and of how phenomenological ideas support and supplement perspectivism, cf. Berghofer (2020a).

18 Husserl (1970, p. 51).

19 Following pioneering works such as the ones by Heelan (1988), Mormann (1991), Bitbol (1996) and French (2002) or Ryckman (2005), recent years have seen a steady increase in studies dealing with the various connections between phenomenology and the physical sciences. For an overview, see Berghofer and Wiltsche (2020). This positive trend notwithstanding, however, still more work needs to be done in order to arrive at a more adequate understanding of how phenomenology and physics are related to each other, both historically and systematically.

20 We emphasize that “represents” has a pragmatic connotation—the quantum state *represents* a physical state for the practical purposes of prediction. The notion of a “physical state” is, in turn, carried over from classical physics, and expresses the view that it is meaningful to speak of the “state” of a body at an instant of time.

21 By “disturbance” we are referring to the fact that it is an intrinsic feature of the quantum formalism that projective measurements necessarily change the observed quantum state. This is why there is a long history of calling a quantum measurement a process of “disturbance” (cf., e.g., Jaeger 2015). However, we want to emphasize that employing this terminology does not mean that we side, e.g, with Bohr’s distinctive view of disturbance. We only refer to the fact that, according to the quantum formalism, measurements change the quantum state, which is uncontroversial. We are thankful to an anonymous reviewer of this journal for pressing us on making this clarification.

22 If the quantum state is written $\psi = Re^{iS/\hbar }$, and the state of the particle is $(\mathbf {x}, \mathbf {p})$, then the guidance equation reads $\mathbf {p} = \nabla S /m$. The particle position is then assumed to evolve classically, so that $\mathbf {x}(t + dt) = \mathbf {x}(t) + \mathbf {p}/m \, dt$.

23 Bohr (1937).

24 Schrödinger (1935).

25 These assumptions are: (1) an experimenter’s choice of measurement can be freely chosen, in particular not being influenced by the initial quantum state of the system; and (2) there is no "conspiracy" in the sense that the initial quantum state of the system is not affected by the experimenters’ later choice of which measurements to perform.

26 Bell (1964).

27 For accessible introductory surveys on the interpretations and implications of quantum theory, the reader is referred to Norsen (2017) and Maudlin (2019).

28 One reviewer has remarked that the temptation to reify mathematical structures is by no means special to quantum theory. We agree. As we have argued earlier, Husserl’s point in §9 of the *Crisis* is that the reification of mathematical idealities becomes part of scientific practice already in the 17th century and thus long before the advent of quantum physics. However, what still makes quantum theory a special case in this discussion is, among other things, that the mathematics operating at its core is much further removed from simple lifeworld intuitions than the mathematical structures featured in classical physics. While it is somewhat understandable that many took Galileo’s geometrical models as truthful representations of dropping stones and flying cannonballs, a straightforward reification seems far less natural in the case of, say, Hilbert spaces. This, in and by itself, is of course no argument for or against any particular understanding of quantum physics. It shows, however, that the issue of mathematization is particularly pressing in this context.

29 Griffiths (2018, p. 94).

30 E.g. Carroll and Singh (2019).

31 Wallace (2013, p. 216).

32 Albert (1996, p. 277).

33 Wallace (2013, p. 217).

34 Wallace (2013, p. 217).

35 Chen (2019, p. 6).

36 Husser (1970, p. 44).

37 Fuchs (2019, p. 136).

38 Chiribella (2016, p. 3).

39 Masanes (2013, p. 16373).

40 Fuchs (2016, p. 285).

41 See Goyal (2020) for a recent, systematic derivation of classical mechanics guided by these fundamental principles.

42 See, for example, Griffiths (2018) for an introduction to the mathematical formalism of quantum theory.

43 See, for example, Hardy (2001), Goyal et al. (2010) and Chiribella et al. (2011).

44 Cf. Husser (1970, §34b).

45 Of course, there is no consensus in the literature on whether quantum information theory could have an important impact on the question of how to interpret quantum theory. For a critical discussion, cf. Timpson (2013). Unfortunately, the project of informationally reconstructing quantum theory has been largely ignored in the philosophical literature (including Timpson 2013). It would go beyond the scope of the paper to defend the significance of informational principles.

46 Wheeler (1989).

47 For example, those reconstructions described in Hardy (2001), Goyal et al. (2010) and Chiribella et al. (2011).

48 As shown in Goyal (2014), indeterminism follows in an operational framework directly from a small number of assumptions, namely (1) continuity, (2) symmetric transition probabilities, and (3) a measurement with a single coarse-grained outcome does not affect the outcome probabilities of subsequent measurements performed on the system.

49 See, for example, Goyal et al. (2010).

50 See, for example, Chiribella et al. (2011).

51 Husser (1970, p. 49).

52 Husser (1970, p. 49).

53 Husser (1970, p. 49).

54 Husser (1970, p. 49).

55 Cf. Berghofer (forthcoming). For how a phenomenological epistemology can enrich current debates, cf. Berghofer (2020b).

56 (Husserl 1960, p. 66).

57 Husserl (2001, p. 100).

58 Husserl (1989, p. 324).

59 Carroll and Singh (2019).

60 Albert (1996).

61 “If ’*positivism*’ is tantamount to an absolutely unprejudiced grounding of all sciences on the ’positive,’ that is to say, on what can be seized upon originaliter, then *we* are the genuine positivists” Husserl (1982, p. 39).

62 2020.

63 Zahavi (2019, p. 54).

64 Zahavi (2019, p. 28).

65 Cf., for the long story, Berghofer and Wiltsche (2019).

66 Husserl (1960, p. 46).

67 Husserl 1982, p. 94).

68 Husserl (1977, p. 121). A similar remark can be found in Husserl (1973, pp. 116f). It is interesting to see that the phenomenologically minded mathematician, physicist and father of the gauge principle Hermann Weyl basically makes the same claim, explicitly drawing on a phenomenological terminology [cf., for further details, (Ryckman 2005, 131 and Wiltsche forthcoming)].

69 Husserl (1969, p. 232).

70 Moran (2012, p. 52).
